# “Paralysis” of “Voluntary” Movement

**Published:** 1918-10

**Authors:** 


### NEUROLOGY AND PSYCHIATRY

---

**The Physiology of " Functional Paralysis " of " Voluntary " Movement.** By T. G. Brown and R. N. Stewart. *The Lancet*, May, 18, 1918.

The authors deal with the functional and psychic paralyses of the limbs, etc., among soldiers who have undergone great stress. A case seen within one hour of onset of functional paralysis and followed for thirty-five days is described in detail. Is was noted that the functional paralysis did not appear immediately after the shock, but a slowly spreading numbness was observed. It was only when the limb was completely numb that the paralysis rather suddenly appeared. The paralysis was at first flaccid — later rigid. Kinesthetic sensations were necessary for the return of " voluntary " power, and when these returned voluntary movements were quickly resumed. At certain joints, however, there was still a failure of appreciation of certain movements.

The authors attempt to define " functional paralysis " and to explain its physiology, in order, as they say, to rationalize the treatment of these cases. They classify the functional disorders into two types, sensory and motor — the former including the functional anesthesias or hyperesthesias, analgesias or hyperalgesias, and the functional abolition or depression of the kinesthectic sensations — functional disturbances of vision, hearing, smell, etc. Likewise there are the functional paralyses of the limbs, aphonia, mutism, etc.

They state categorically that the " functional paralyses " of a limb very often, if not always, are accompanied by functional disturbance of sensation — anesthesia or analgesia, or both; functional aphonia, by anesthesia or hyperesthesia of the mucous membrane of the larynx or pharynx; and mutism is often accompanied by deafness.

The fact that the monkey, in which a limb is desensitized, makes no voluntary use of that limb is exactly comparable, they argue, to the functional paralysis.

The motor mechanism of the cerebral cortex becomes inactive in that part of it which is cut off from the sensory (centripetal) nerve impulses of the part of the body which it subserves, but the mechanism itself remains a workable one; i. e., there is probably a sort of " cortical tone " which is kept at a suitable pitch by centripetal impulses which play upon the mechanism.

The authors suggest that the cause of functional paralyses, so-called, is secondary to the anesthesias (in the widest sense of the term) of the parts affected. In support of this they say : " Just as in the monkey functional paralysis of a limb follows the destruction of the afferent nerves of the limb, so, in man, a functional paralysis follows a depression of the sensations of the paralysed part of the body, especially if this depression affects the self-engendered sensations ".

The fact that the functional paralysis tends often to persist after the resolution of the anesthesia is explained by them by the long disuse of the cortical mechanism, making it relatively inexcitable to the impulses which normally play upon it.

Their explanation of the functional gaits and rigidities is as follows : Cortical reactions are characterized by their *phasic* nature (in which the angles between the bones of a joint are constantly changing) occasioned by changing degrees of contraction and relaxation of the muscle of the limb, e. g., the progression in the water of a man swimming. Reactions of the red nucleus are characterized by their *postural* nature (in which there are no movements of the bones relative to each other at the joints, but where the limb is held in a certain posture by a maintained contraction of the muscles), e. g., the slow, difficult progression of a man bearing a heavy weight where the limbs are used as props with a minimum of movement. These facts were proven by the electrical stimulation of the cerebral cortex and of the red nucleus respectively.

Progression is fundamentally a spinal activity — modified in its phasic direction by the activity of the cortical mechanism, and in the postural direction by the red nuclear mechanism. " Functional Paralysis ", fully developed, is probably due to an abnormal exaggeration of the normal manner (postural) of holding and moving the limbs, which is facilitated by use and conditioned by a a relative difficulty in activating the cerebral (phasic) mechanism. The relative difficulty is, in its turn, conditioned by the failure of certain centripetal sensory impulses to rise above the threshold of the cortical motor mechanism. The " postural " state is a " bad habit ".

Therefore on rational grounds it is best to treat the condition as early as possible and before this abnormal state has appeared or has become fixed or facilitated. The primary condition is the anesthesia, which, when resolved, allows impulses to play upon the cortical mechanism which are ineffective in the state of functional paralysis — the gait, posture, etc.

